# Role of PTEN-less in cardiac injury, hypertrophy and regeneration

**DOI:** 10.1186/s13619-021-00087-3

**Published:** 2021-08-02

**Authors:** Tian Liang, Feng Gao, Jinghai Chen

**Affiliations:** 1 Department of Cardiology, Provincial Key Lab of Cardiovascular Research, Second Affiliated Hospital, Zhejiang University School of Medicine, Hangzhou, 310009 Zhejiang China; 2 Institute of Translational Medicine, Zhejiang University School of Medicine, Hangzhou, 310009 Zhejiang China

**Keywords:** PTEN, Cardiac hypertrophy, Cardiomyocytes proliferation, Regeneration, Cardiac apoptosis

## Abstract

Cardiovascular diseases are the leading cause of death worldwide. Cardiomyocytes are capable of coordinated contractions, which are mainly responsible for pumping blood. When cardiac stress occurs, cardiomyocytes undergo transition from physiological homeostasis to hypertrophic growth, proliferation, or apoptosis. During these processes, many cellular factors and signaling pathways participate. PTEN is a ubiquitous dual-specificity phosphatase and functions by dephosphorylating target proteins or lipids, such as PIP3, a second messenger in the PI3K/AKT signaling pathway. Downregulation of PTEN expression or inhibiting its biologic activity improves heart function, promotes cardiomyocytes proliferation, reduces cardiac fibrosis as well as dilation, and inhibits apoptosis following ischemic stress such as myocardial infarction. Inactivation of PTEN exhibits a potentially beneficial therapeutic effects against cardiac diseases. In this review, we summarize various strategies for PTEN inactivation and highlight the roles of PTEN-less in regulating cardiomyocytes during cardiac development and stress responses.

## Background

Cardiovascular diseases are the leading cause of mortalities and affects more than 26 million people (Roth et al. 2017; Bui et al. 2011). Myocardial injury causes enormous amount of cardiomyocytes loss, resulting in compromised cardiac contraction and pathological cardiac dilatation, accompanied with cardiac compensatory hypertrophic and fibrotic remodeling in hearts. Due to the limited proliferation capacity of mature cardiomyocytes, the damaged heart hardly gets regeneration and enough repair. Despite the significant progress in clinical treatment of cardiac diseases, morbidity and mortality rates remain high (Roth et al. 2017). An alternative strategy for treatment of cardiac diseases is promoting cardiac endogenous repair by regulating cardiomyocytes proliferation and cellular biological processes in regeneration, which rely on essential signal pathway cascades.

PTEN (phosphatase and tensin homolog), also known as MMAC1 (mutated in multiple advanced cancers) or TEP1(TGFb-regulated and epithelial cell-enriched phosphatase), was first identified as a tumor suppressor gene in 1997 by three independent groups through mapping human homozygous deletion on chromosome 10q23 (Li et al. 1997; Steck et al. 1997; Li and Sun 1997). PTEN mutation occurs frequently in multiple human advanced cancers, such as brain, breast, prostate cancer and glioblastomas (Li et al. 1997; Steck et al. 1997; Li and Sun 1997). PTEN acts as a dual-specificity phosphatase that dephosphorylates lipids and proteins on serine, threonine and tyrosine residues (Myers et al. 1997). Overexpression of PTEN inhibits tumor growth and cell migration by reducing the tyroshine phosphorylation of focal adhesion kinase FAK (Tamura et al. 1998). To evaluate the roles of PTEN in oncogenesis in vivo, researchers generated conventional *Pten* knockout mouse in 1998 by removing exons 4 to 5, or exons 3 to 5 of the *Pten* gene in ES cells (Di Cristofano et al. 1998; Suzuki et al. 1998; Stambolic et al. 1998). *Pten* ablation resulted in early embryonic lethality, implying that PTEN is an essential factor in embryonic development (Di Cristofano et al. 1998; Suzuki et al. 1998). In addition, PTEN negatively regulates cellular phosphatidylinositol(3,4,5) trisphosphate (Ptdlns(3,4,5)-P_3_) and dephosphorylates it, which is an activator of 3-phosphoinostide-dependent kinease (PDK) and AKT. Thus, PTEN functions as a tumor suppressor by negatively regulating PI3K/AKT signaling pathway (Stambolic et al. 1998). The crystal structure of human PTEN revealed the overall structure of PTEN and the binding site of PTEN with Ptdlns(3,4,5)-P_3_, which provides further evidence for the above conclusion (Lee et al. 1999).

In the past two decades, researchers have unveiled the crucial role of PTEN in development, tumorigenesis, as well as in heart growth. As a ubiquitous gene, *Pten* is widely expressed in many tissues and cells including the heart and cardiomyocytes. Using a muscle-specific *Pten* knockout mouse model, Josef M. Penninger group found PTEN inactivation promotes heart hypertrophy and decreases cardiomyocyte contractility (Crackower et al. 2002), indicating PTEN plays a fundamental role in cardiac physiology. Noticeably, under pathological stimuli, loss of PTEN results in marked and persistent protection against aortic banding-induced stress (Oudit et al. 2008). Since PTEN negatively regulates PI3K/AKT while activation of Akt protects cardiomyocytes from apoptosis and heart function from cardiac injury (Fujio et al. 2000), inactivation of PTEN emerged as a potential therapeutic method against cardiac diseases, especially ischemic cardiac stress (Oudit et al. 2008; Ruan et al. 2009). Actually, the roles and underlying mechanisms of PTEN in regulation of cardiac physiological and pathological processes, have attracted much attention in heart research over years.

In this review, we use the word ‘PTEN-less’ to refer to PTEN loss or inactivation (Stiles et al. 2004), and we summarize the roles of PTEN-less in common basic biological processes of cardiomyoccytes in diseased heart, such as hypertrophy, proliferation, apoptosis and survival. We anticipate to increase understanding of the function and mechanism of PTEN-less in cardiomycytes fate, and to promote the gene therapy development in heart regeneration field.

## Approaches to PTEN inactivation

Regulation of PTEN expression and PTEN activity is achieved through various methods, including genetic, post-transcriptional and post-translational mechanisms.

### Genetic regulation

The first transgenic mouse harboring loss-of-function mutation in *Pten* gene was generated in 1998 by replacing exons 4 and 5 of *Pten* gene with the neomycin-resistance gene (neo) cassette, resulting in a functionally inactive *Pten* allele (Di Cristofano et al. 1998). Around the same time, another research group also created a similar *Pten* mutant mouse line. They generated *Pten*^−/−^ mice through targeted deletion of exons 3 to 5 of *Pten* gene (Suzuki et al. 1998). These two lines of conventional *Pten*^−/−^ mice lead to early embryonic lethality, indicating that conditional *Pten* knockout mice are needed for deeper mechanistic studies.

Mice with conditional mutagenesis of *Pten* gene were first generated in 2001 by two groups. Suzuki et al. used the Cre-loxP system (expressing Cre recombinase under control of the *Lck* promoter) to generate a T cell-specific deletion of the *Pten* gene by targeting exons 4 and 5*.* Mice with heterozygous deletions of *Pten* were born alive and appeared healthy (Suzuki et al. 2001). Based on this floxed *Pten* mice, conditional *Pten* knockout mice were generated using different tissue specific Cre, such as *Gfap-Cre* (brain) (Backman et al. 2001), *Mck-Cre* (heart and skeletal muscle) (Crackower et al. 2002), *Alb-Cre* (hepatocyte) (Horie et al. 2004) and *Nse-Cre* (neurons) (Kwon et al. 2006). Another different line of *Pten* floxed mouse was generated nearly at the same time. LoxP sequences were inserted into the endogenous *Pten* locus flanking exon 5, which encodes the phosphatase domain and accounts for many tumor-associated mutations. *Pten*^*flox/flox*^ mice can be born with normal PTEN expression levels (Lesche et al. 2002; Groszer et al. 2001). To verify Cre recombinase-induced deletion of *Pten* exon 5, they crossed *Pten*-floxed females with males carrying a nestin promoter-driven *Cre* transgene which is activated in central nervous system stem/progenitor cells at embryonic day (E) 9 or 10. There are no PTEN expresision in whole brain lysates from newborn *Pten* mutant mice (Groszer et al. 2001). This *Pten* floxed mouse line was also widely applied in the heart. Ruan et al. established a mouse genetic model of cardiomyocyte specific and tamoxifen inducible ablation of *Pten* to investigate the functional role of PTEN in response to ischemia/reperfusion (Ruan et al. 2009). Liang et al. used tamoxifen inducible cardiomyocyte specific *Pten* knockout mice to investigate the role of *Pten* in cardiac regeneration after myocardial infarction (Liang et al. 2020). Liu et al. used AAV-Cre to induce *Pten* deletion, and found that deletion of *Pten* enhanced compensatory sprouting of uninjured corticospinal tract axons and enabled regeneration of a cohort of injured corticospinal tract axons past a spinal cord lesion (Liu et al. 2010).

The summary of *Pten* knockout mice is listed in Table 1.

**Table 1:** *Pten* knockout mice

Conventional Knockout						
Pten Exons		**Target cells**	**Effect**	**Years**	**Ref.**	
Exons 3–5		ES cells	Embryonic lethality	1998	(Suzuki et al. 1998)	
Exons 4–5		ES cells	Embryonic lethality	1998	(Di Cristofano et al. 1998)	
Conditional Knockout						
Floxed Exons		**Tissue specific deletion**				
Pten Exons	**Ref.**	**Cre**	**Target cells**	**Effect**	**Years**	**Ref.**
Exons 4–5	(Suzuki et al. 2001)	*Lck-Cre*	T cells	*Pten* knockout T cells are autoreactive, hyperproliferate, resist apoptosis and secreate high level Th1/Th2 cytokines, show increased p-PKB/Akt and p-ERK	2001	(Suzuki et al. 2001)
Exons 4–5		*Gfap-Cre*	Brain glial cells	Mice showed enlarged brain and developed seizures and ataxia by 9 weeks and died by 29 weeks, *Pten* mutant cells shoewed an increased soma size and elevated p-Akt	2001	(Backman et al. 2001)
Exons 4–5		*Mck-Cre*	Skeletal and cardiac muscle	Knockou *Pten* induced heart hypertrophy without pathlogical change and decreaed heart contractility through mediating PI3Kγ	2002	(Crackower et al. 2002)
Exons 4–5		*Alb-Cre*	Hepatocyte	Mice showed massive hepatomegaly and steatohepatitis with triglyceride accumulation, hepatocytes showed hyperproliferation and abnormal activation of protein kinase B and MAPK	2004	(Horie et al. 2004)
Exons 4–5		*Nse-Cre*	Differentiated neurons in the cerebral cortex and hippocampus	Mice showed abnormal social interaction and exaggerated responses to sensory stimuli, with activation of the Akt/ mTor/S6k pathway and inactivation of Gsk3β	2006	(Kwon et al. 2006)
Exons 4–5		*Mck-Cre*	Skeletal and cardiac muscle	Mice showed reduced pathological hypertrophy, less interstitial fibrosis, reduced apoptosis and marked preservation of LV function in aortic banding induced pressure overload model, and markedly reduced p-JNK1,p-JNK2 and p-p38	2008	(Oudit et al. 2008)
Exons 4–5		*SM22α-Cre*	Smooth muscle cells	Mice shopwed widespread medial SMC hyperplasia, vascular remodeling, and histopathology consistent with pulmonary hypertension	2008	(Nemenoff et al. 2008)
Exons 4–5		*PdgfbiCreER*^*T2*^ (Cre induced by tamoxifen)	Endothelial cell	Endothelial deletion of PTEN results in vascular hyperplasia because cannot regulate Notch-induced proliferation arrest. Both the catalytic and non-catalytic APC/C-Fzr1/Cdh1-mediated activities of PTEN are required for stalk cells’ proliferative arrest	2015	(Serra et al. 2015)
Exon 5	(Lesche et al. 2002; Groszer et al. 2001)	*Nestin-Cre*	Central nervous system stem/progenitor cells	Mice deletion PTEN showed enlarged and abnormal brains, with increased cell proliferation, decreased cell death, and enlarged cell size	2001	(Groszer et al. 2001)
Exon 5		*ARR2Probasin-Cre*	Prostatic epithelial cells	*Pten* deletion successfully induced murine prostate cancer model	2003	(Wang et al. 2003)
Exon 5		*Mx-1-Cre* (Cre induced by polyinosine-polycytidine)	Bone marrow Haematopoietic stem cells (HSCs)	The ability of sustain haematopoietic reconstitution affected in *Pten*-deicient HSCs, mice with *Pten* deletion showed an increased representation of myeloid and T-lymphoid lineages and develop myeloproliferative disorder	2006	(Zhang et al. 2006)
Exon 5		*Gdf-9-Cre*	Oocytes	Lacking PTEN in oocytes activated the entire primordial folliclepool and caused premature ovarian failure	2008	(Reddy et al. 2008)
Exon 5		*α-MHC-MerCreMer* (Cre induced by tamoxifen)	Cardiomyocytes	PtenCKO hearts exhibited increased PI3K activity in baseline,and better function recovery after ischemia/reperfusion,with fewer apoptosis and higher level of ERK and BCL-2 expression	2009	(Ruan et al. 2009)
Exon 5		AAV- Cre	Corticospinal neurons	Deletion PTEN enhanced compensatory sprouting of of uninjured corticospinal tract axons and enabled regeneration of a cohort of injured corticospinal tract axons past a spinal cord lesion	2010	(Liu et al. 2010)
Exon 5		*Pax7*^*CreER*^	Quiescent satellite cells	Quiescent satellite cells specific knockout *Pten* lead to spontaneous activation and premature differentiation and resulted in failed regeneration. Mechanistically, *Pten* deletion increases Akt phosphorylation, further induced FoxO1 cytoplasmic translocation and Notch signalling suppression	2017	(Yue et al. 2017)
Exon 5		*α-MHC-MerCreMer* (Cre induced by tamoxifen)	Cardiomyocytes	Cardiac-specific knockout *Pten* in adult mice preserved heart function, decreased scar size and promoted cariomyocytes proliferation after myocardial infarction stress	2020	(Liang et al. 2020)

### Post-transcriptional regulation

MicroRNA is a commonly used strategy for post-transcriptional regulation. Due to relatively long 3′ untranslated region (UTR) sequence, *Pten* mRNA can be easily targeted by many microRNAs, such as microRNA-19a, microRNA-19b (Chen et al. 2013) and microRNA-301a (Zhen et al. 2020), resulting in downregulated expression level. Therefore, post-transcriptional regulation of PTEN expression by noncoding RNAs, especially, microRNAs, is frequently involved in modulation of pathophysiological processes during development, homeostasis, and disease.

### Post-translational regulation

For post-translational regulation, small molecule inhibitors are generally and widely applied in translational therapy. Protective effects of the PTEN inhibitor on cardiac functions were first reported in 2010, when researchers showed that suppression of PTEN by bisperoxovanadium molecules [BpV (HOpic)] decreased mice myocardial infaction size and improved heart function post ischemia/reperfusion injury (Keyes et al. 2010). In addition, *Pdk1*-deficient mice exhibited heart dilation and failure, however, treatment with PTEN inhibitor bpV (phen) prolonged mice survival by enhancing Akt Ser473 phosphorylation (Zhao et al. 2014). Noticeably, PTEN heterogeneity is carcinogenic and inhibition of PTEN by pharmacological methods enhances tumor growth (Xi and Chen 2017).

As PTEN is a member of the large family of cysteine-based phosphatases (CBPs) that contains the protein tyrosine phosphatase (PTPase) superfamily, some well-established general PTPase inhibitors, such as vanadium and peroxovanadium compounds, inhibit PTEN activity and also inhibit a broad range of phosphatases (Huyer et al. 1997; Posner et al. 1994). To design and synthesize specific vanadium-based PTEN inhibitors, Rosivatz et al. synthesized eight small recombinant vanadium compounds, including VO-OHpic, bpV-OHpic, bpV-pic, VO-pic, bpV-biguan, VO-biguan, bpV-phen, and bpV-isoqu. These compounds are shown to bind to the active site of PTEN but show little activity against other PTPases (Rosivatz et al. 2006). After comparing these eight compounds against enzyme activities of four other recombinant CBPs (PTP-β, SAC1, MTM1 and SopB) in vitro, they found VO-OHpic is the most potent and specific inhibitor for PTEN, whereas the other vanadium compounds possess broader specificity (Rosivatz et al. 2006). In addition, SF1670, a phenanthrenedione-related compound, is also used as a relatively specific PTEN inhibitor. Pretreated with SF1670 in neutrophils enhanced the inflammatory response and the bacteria-killing capability in neutropenic recipient mice (Li et al. 2011). A summary of the role of PTEN specific inhibitors in various biological systems are shown in Table 2.

**Table 2:** PTEN specific inhibitors

Compound	Dose	Object	Effect	Years	Ref.
SF1670	125-500 nM	Human and mouse neutrophils	Neutrophils treatment with PTEN specific inhibitor SF1670 elevated Ptdlns(3,4,5)-P_3_ signaling, enhanced the innate immune responses. Mice transfusion with SF1670-treated neutrophils led to augmented bacteria-killing capacity in both peritonitis and bacterial pneumonia	2011	(Li et al. 2011)
SF1670	10uM	Human colorectal cancer (CRC) cell lines	Selenite could induce FoxO3a-mediated apoptosis in CRC cells through PTEN-regulated AKT/FoxO3a/Bim signaling pathway, inhibition PTEN by SF1670 abrogated the above changes	2013	(Luo et al. 2013)
SF1670	3 nM	Neuronal progenitor striatal cells (NPC) from mouse striatum	Inactivation of PTEN in NPC with SF1670 enhanced the inhibition effect of BDE-49 on mitochondrial respiratory chain electron transport	2013	(Napoli et al. 2013)
SF1670	10uM	Human pre-B acute lymphoblastic leukemia (ALL)	Inhibition of PTEN with SF1670 in human pre-B ALL cells induced cell death, with hyperactivation of AKT and activation of the p53 tumor suppressor cell cycle checkpoint	2016	(Shojaee et al. 2016)
VO-OHpic	500 nM	Human prostate cancer cell lines	Inhibition PTEN with VO-OHpic induces senescence and inhibits tumorigenesis in prostate cancer through enhance p53 translation	2010	(Alimonti et al. 2010)
VO-OHpic	500 nM	Carcinoid cell line BON	Inhibition of PTEN with VO-OHpic in BON cells result in decreased secretion and synthesis of serotonin,with increased Akt signaling	2011	(Silva et al. 2011)
VO-OHpic	100 nM	Mouse adrenal chromaffin cells	Abolished the effect of PI3Kδ inhibitor IC87114 on promoting potentiation of Ca^2+^ − stimulated catecholamine release	2011	(Wen et al. 2011)
VO-OHpic	500 nM	Breast cancer cells	Abolished the effect of PI3Kδ inhibitor IC87114 on AKT inhibition	2012	(Tzenaki et al. 2012)
VO-OHpic	0.1-5uM	Mouse ventricular cardiomyocytes	Highly protective against cell death induced by ischemia and reperfusion	2014	(Zhu et al. 2014)
VO-OHpic	10 μg/kg BW	Mice	Induce cooling-like protection with improved recovery and survival in mouse model of SCA	2015	(Li et al. 2015)
VO-OHpic	500 nM	Human hepatocellular carcinoma cell lines	Inhibited cell growth and induced senescence in hepatocellular carcinoma (HCC) cells	2016	(Augello et al. 2016)
VO-OHpic	100uM	Mice superior mesenteric artery	Improved insulin-induced vasodilation in high fat diet-fed mice	2016	(da Costa et al. 2016)
VO-OHpic	500 nM	Mouse OS tumor cells and human OS cell line	Facilitate tumor growth and expansion in bone	2017	(Xi and Chen 2017)
VO-OHpic	0.05/2 μg/ml	Rat cardiac myocytes	Improves cardiac myocyte survival after ischemic reperfusion by mediating apoptosis resistance in vitro	2018	(Zhang et al. 2018)
VO-OHpic	10 μg/kg BW	Mice	Preserve heart function and promote cardiomyocytes proliferation after myocardial infarction	2020	(Liang et al. 2020)

## PTEN in cardiac hypertrophic growth

Cardiac hypertrophy, a common pathophysiological phenomenon, occurs during exercise, pregnancy, and in many cardiac diseases, such as hypertension, ischemic heart disease, valvular disease and heart failure (Nakamura and Sadoshima 2018; Frey et al. 2004). The heart initiates proceeds hypertrophic growth in response to hemodynamic overload to increase contractility and diminish ventricular wall stress. However, this adaptive compensation eventually leads the hypertrophic heart transition to heart failure through pathological remodeling, characterized by an increased cardiomyocyte size and enlarged heart volume (Nakamura and Sadoshima 2018; Frey et al. 2004). Cardiac hypertrophy is regulated by multiple signaling pathways, including PI3K/AKT, which play crucial roles in regulation of cell growth, cell survival, and metabolism (Crackower et al. 2002; Oudit et al. 2003). There are three classes (I-III) of PI3K. Primarily, activated PI3K (Class I) phosphorylates phosphatidylinositol-4,5-bisphosphate (PIP2) and converts PIP2 to phosphatidylinositol-3,4,5-trisphosphate (PIP3), and subsequently activates downstream Akt signaling (Engelman et al. 2006). Class IA PI3K, consisting of a regulatory subunit and a p110(α, β, δ) catalytic subunit, are activated by growth factor receptor tyrosine kinases. Class IB PI3K, consisting of a regulatory subunit and a p110γ catalytic subunit, are activated by G-protein-coupled receptors (Engelman et al. 2006). Cardiac-specific expression of constitutively active class IA PI3K(p110 α) increases the cardiomyocytes size, and induces heart hypertrophy in mice. Consistently, expression of dominant negative PI3K(p110 α) reduces cell size of cardiomyocytes with no appearance difference in heart function (McMullen et al. 2003). However, loss of class IB PI3K(p110 γ) improves the cardiac contractility by elevating cAMP levels in mice (Crackower et al. 2002).

PTEN negatively regulates PI3K-AKT signaling by dephosphorylating PIP3, further affecting AKT phosphorylation. Inactivation of PTEN between E6.5 to E9.5 resulted in embryonic lethality in mouse (Suzuki et al. 1998). As to PTEN’s role in heart development, Penninger group knocked out *Pten* in mouse muscles (*Pten*^flox/flox^; *Mck-Cre*). They found heart size increased in the knockout group in 10 weeks and 12 months. Moreover, phosphorylations of GSK3β and p70^S6K^ were increased in the hypertrophic heart induced by *Pten* knockout (Crackower et al. 2002). Thereafter, they used the same genetic mouse model with aortic banding (AB) to mimic hypertension-induced cardiac hypertrophy in humans. The control group (*Mck-Cre*) exhibited a marked ventricular dilation and loss of systolic function in heart post 9- and 12- weeks aortic banding. Intriguingly, the *Pten* knockout group (*Pten*^flox/flox^; *Mck-Cre*) showed a minimal ventricular hypertrophy and dilation, indicating that loss of PTEN protected heart from AB injury (Oudit et al. 2008). Recently, Liang et al. generated cardiac-specific inducible *Pten* knockout mice and performed acute myocardial infarction (MI) on the *Pten*-CKO mice (*Pten*^flox/flox^; *αMHC-MCM*) and control mice (*Pten*^flox/flox^). Similarly, they found cardiac specific deletion of *Pten* significantly decreased cardiomyocytes size at 12 weeks post MI, and consistently preserved heart function from 2 weeks to 12 weeks post MI (Liang et al. 2020). These studies indicate that loss of PTEN attenuates cardiac hypertrophic growth in pathological remodeling and protects heart function after cardiac stress such as aortic banding and myocardial infarction.

## PTEN in cardiomyocyte proliferation and cardiac regeneration

Heart regeneration has attracted more and more attention of researchers since 1850s (King 1940; Carvalho and de Carvalho 2010; Zheng et al. 2021; Cutie and Huang 2021). It is generally believed that lower vertebrates, such as newt and zebrafish, have the ability to regeneration throughout life (Poss et al. 2002; Jopling et al. 2010; Kikuchi et al. 2010; Lepilina et al. 2006). However, the mammalian hearts only have the regenerative ability in embryo and early postnatal stage since the adult cardiomyocytes are considered as terminally differentiated and hardly divide (Kathiresan and Srivastava 2012; Mudd and Kass 2008). After apical resection, the heart of postnatal 1-day-old mice can regenerate with complete functional recovery, but the mice lost the capability of such spontaneous regeneration by 7 days of age (Porrello et al. 2011). In adulthood, mature cardiomyocytes retain limited regenerative capacity with about 1% measurable turnover and increase such capacity by several fold in response to injury (Bergmann et al. 2009; Bergmann et al. 2015; Senyo et al. 2013; Porrello et al. 2013). Using isotope of nitrogen labeling and lineage tracing approach in mouse model, researchers have concluded that the newly generated cardiomyocytes arise from pre-existing cardiomyocytes but not from nonmyocytes (Porrello et al. 2011; Senyo et al. 2013; Li et al. 2018a).

Cardiac diseases, like myocardial infarction, cause the loss of a billion of cardiomyocytes during pathological injury. The key to mend the damaged heart is to regenerate the cardiomyocytes. However, this regeneration capacity of cardiomyocyte is too low to fully recover in heart disease from a regenerative perspective. Finding endogenous stimulation to boost cardiomyocytes proliferation and heart regeneration is critical for treating heart disease. Recently, scientists have discovered several cellular factors regulating cardiomyocytes cell cycle. They found that homeodomain transcription factor Meis1, is required for transcriptional activation of the synergistic CDK inhibitors p15, p16 and p21. Cardiac specific knockout of Meis1 can promote cell cycle activity in young mouse hearts (Mahmoud et al. 2013). Conditional double knockout of Meis1 and its co-factor Hoxb13 have a significant increase in the number of ventricular cardiomyocytes and have a gradual and significant improvement in heart function after myocardial infarction (Nguyen et al. 2020). Besides protein, noncoding RNAs, especially microRNAs, often participate in regulation of cardiomyocyte proliferation and cardiac regeneration during cardiac homeostasis or after heart injury. In an elegant study, Eulalio et al. performed high-throughput functional screening in rodent cardiomyocytes and they identified certain important microRNAs, hsa-miR199a and hsa-miR590a can promote neonatal cardiomyocyte proliferation, and stimulate adult cardiomyocyte re-enter cell cycle and division (Eulalio et al. 2012). More importantly, the same group further demonstrated that in large animal, AAV-mediated overexpression of miR-199a in porcine hearts significantly stimulates cardiomyocytes proliferation and improves heart function after injury from myocardial infarction (Gabisonia et al. 2019).

For post-transcriptional regulation, Chen et al. have demonstrated that miR-17-92 cluster is required for cardiomyocyte proliferation in the mouse heart (Chen et al. 2013). Cardiac specific overexpression of miR-17-92 with miR-17-92-KI mouse is sufficient to stimulate cardiomyocyte proliferation in embryonic, postnatal and adult hearts (Chen et al. 2013). The expression of PTEN is inversely correlated with the expression of miR-17–92, which is decreased in the hearts of miR-17–92 cardiac knock-in mice and increased in miR-17–92 cardiac knockout mouse heart (Chen et al. 2013). *Pten* has benn reported as a direct target of miR-19 family (miR-19a/19b), which are the most potent members of the miR-17-92 cluster (Olive et al. 2009). Overexpression miR-19a/19b by intra-cardiac injection of miRNA mimics is capable to stimulate cardiomyocytes proliferation and repairs the adult heart after myocardial Infarction with downregulated PTEN expression level (Gao et al. 2019), whereas overexpression of PTEN reverses miR-19-induced proliferation in cultured cardiomyocytes (Chen et al. 2013). Interestingly, overexpression mir-17-3p (a passenger miRNA of miR-17, which is a member of miR-17-92 cluster) through tail vein injected miRNA agomir also promotes cardiomyocytes proliferation and decreases expression of PTEN indirectly in isolated neonatal rat cardiomyocytes (Shi et al. 2017).

MiR-301a is specially enriched in the neonatal cardiomyocytes of rats and mice. Overexpression of miR-301a in mice through tail vein injected AAV9 virus improves heart function, promotes cardiac repair as well as myocardium regeneration, and decreases cardiac fibrosis after myocardial infaction. *Pten* has been found to be a target gene of miR-301a in cardiomyocytes. Down regulation of *Pten* is accompanied with increased expression of p-AKT and p-GSK3β in miR-301a treated mouse heart, indicating PTEN/PI3K/AKT signaling pathway mediates the cardiac regeneration induced by miR-301a (Zhen et al. 2020).

In addition, a novel lncRNA AZIN2-sv (splice variant), highly expressed in adult heart, negatively regulates endogenous cardiomyocyte proliferation of SD rats in vivo and in vitro. Knockdown of AZIN2-sv with shRNA adenovirus attenuates ventricular remodeling and improves cardiac function after myocardial infarction. AZIN2-sv acts as a microRNA-214 sponge to release *Pten*, which in turn blocks activation of the PI3K/Akt signal pathway and inhibits cardiomyocyte proliferation (Li et al. 2018b).

Although cardiomyocytes proliferation and regeneration regulated by noncoding RNAs appears to associate with PTEN inhibition, the convincing evidence that PTEN inactivation directly stimulates cardiomyocytes proliferation is missing until recent report (Liang et al. 2020). Liang et al. generated cardiac-specific knockout of *Pten* mice with a tamoxifen-inducible Cre-loxP system (*Pten*-cKO) and subjected the mice to myocardial infarction injury to study the cardiac regeneration. Using in vivo genetic approach, Liang et al. demonstrated that cardiac knockout of *Pten* promotes cardiomyocytes proliferation, reduces cardiac hypertrophy and infarcted area, and improves heart function after myocardial infarction. The regenerative phenomena in heart of *Pten*-cKO mice post injury was further confirmed when they employed an independent lineage tracing strategy using R26R-Confetti Cre-reporter system with loxP-flanked multicolor fluorescent proteins (nuclear green fluorescent protein (nGFP), red fluorescent protein (RFP), yellow fluorescent protein (YFP) and monomeric cyan fluorescent protein (mCFP)). A small number of cardiomyocytes randomly express one of four fluorescent proteins induced by low dose tamoxifen, the same color adjacent cardiomyocytes are generated by cell proliferation most likely (Snippert et al. 2010; Wang et al. 2017). More clinically relevant, they additionally demonstrated that PTEN inhibitor, VO-OHpic at even very low dose, also protects heart function and structure from myocardial infarction injury and boosts cardiac regeneration (Liang et al. 2020), which may be a therapeutic strategy for ischemic heart disease.

In other organs, such as the central nervous system, PTEN signaling has been shown to be involved in cell regeneration. The ability of regeneration in injured axons declines with age. The biggest challenge in the adult central nervous system is adult axons lose the ability to regeneration and often need to travel long distances to reconnect with their targets (Schwab and Bartholdi 1996). Mammalian target of rapamycin (mTOR) pathway is suppressed in adult central nervous system, reactivating the mTOR pathway by silencing PTEN in adult retinal ganglion cells can induce extensive axon regeneration (Park et al. 2008). The regrowth ability of corticospinal tract (CST) axons lost after development for the low mTOR activity in mature corticospinal neurons. Conditional knockout of *Pten* with injected AAV-Cre into the corticospinal neurons of *Pten*^flox/flox^ mice sustains a high level of mTOR activity, and induces regeneration of a cohort of injuryed CST axons past a spinal cord lesion. The regenerating CST axons from *Pten* deletion seems to have the capability of reforming synapses in caudal segments (Liu et al. 2010). In addition, deletion of the suppressor of cytokine signaling 3 (SOCS3) in adult retinal ganglion cells (RGCs) elicited a robust regeneration of injured optic nerve axons (Smith et al. 2009). However, this two strategy could only maintain regeneration capacity for two weeks after optic nerve injury. For long term stimulation, researchers simultaneously deleted both PTEN and SOCS3, and found co-deletion of PTEN and SOCS3 triggered robust and sustained axon regeneration through regulating activation of mTOR and STAT3 pathway (Sun et al. 2011). Mechanistically, alpha-retinal ganglion cells (aRGCs) accounts for the regeneration following down-regulation of PTEN with high level of mTOR activity. The aRGCs selectively express osteopontin (OPN) and receptors for the insulin-like growth factor 1 (IGF-1). Administration of OPN and IGF-1 induce regeneration similar as PTEN deletion (Duan et al. 2015).

## PTEN in cardiomyocyte apoptosis and survival

In addition to cardiac regeneration, preventing cardiomyocytes apoptosis and promoting their survival are very important for heart repair after myocardial injury in diseases. Loss of PTEN suppresses cell apoptosis and promotes cell survival through activating the PI3K/AKT signaling pathway (Mocanu and Yellon 2007; Wu et al. 2006). PI3K/AKT signal pathway is the main pro-survival pathway, activation of the PI3K/AKT pathway protects the heart from ischemia-reperfusion injury (Cai and Semenza 2005; Hausenloy and Yellon 2004; Rossello et al. 2018). Given the negative correlation between PTEN and PI3K/AKT signaling pathway, loss of PTEN becomes a potential therapeutic target for increasing myocardial survival against cardiac stress injury (Oudit et al. 2004).

Through transgenic mice, it was found that cardiac-specific knockout of *Pten* protects heart from ischemia/reperfusion injury by enhancing the expression of anti-apoptotic gene Bcl-2 and pro-survival signaling ERK (Ruan et al. 2009). Transgenic hearts with cardiac-specific overexpression of miR-494 displays better functional recovery under ischemia/reperfusion injury. In addition, overexpression miR-494 in cultured adult cardiomyocytes reduces caspase-3 activity. The miR-494 target genes ROCK1, PTEN, CAMKIIδ, FGFR2, and LIF are involved in regulating the p-Akt mediated apoptosis signaling (Wang et al. 2010).

Furthermore, intra-myocardially injected miR-19a/19b mimics in myocardial infaction mice preserves heart function, decreases PTEN expression and inhibits apoptosis with reduced TUNEL and cleaved caspase 3 levels (Gao et al. 2019). Overexpression of miR-130a through injecting lentivirus into mice myocardium protects heart from myocardial infarction injury and decreases PTEN expression levels, but whithout affecting apoptosis (Lu et al. 2015). From in vitro studies, transfection of miR-19a mimic inhibites PTEN expression, increases p-Akt levels, attenuates H9C2 cardiomyocytes apoptosis and decreases LDH release under hypoxia/reoxygenation(H/R) (Sun et al. 2017). Overexpression of miR-19b using mimic in H9C2 cells decreases PTEN expression, improves cell survival and decreases apoptosis induced by H_2_O_2 (__Xu et al._ 2016_)_. MiR-885 mediates cardio-protection against hypoxia/reoxygenation-induced apoptosis, and reduces the levels of cleaved caspase-3 and -9 proteins in human cardiomyocytes via inhibiting PTEN and BCL2L11 by modulating AKT/mTOR signaling (Meng et al. 2020).

From a post-translational regulation view, inhibiton of PTEN by a specific inhibitor, VO-OHpic, protects heart tissue by apoptosis resistance after ischemic stress, recovers the heart function, and decreases myocardial infarcted size after ischemia reperfusion (Zhang et al. 2018; Zu et al. 2011). Administration of another PTEN inhibitor bisperoxovanadium (BpV) in rat cardiomyocytes subjected to ischemia/reperfusion protects them from simulated ischemia/reperfusion injury through up-regulating the PI3K/AKT/eNOS/ERK pro-survival pathway (Keyes et al. 2010).

From bench to bedside, a novel clinical combination drug, Sacubitril/Valsartan (Brand name Entresto®), has been proved superiority over conventional heart failure medical treatments in reducing cardiomyocyte cell death, hypertrophy, and improving myocyte contractility by inhibiting PTEN (Iborra-Egea et al. 2017). Additionally, the traditional Chinese medicine Baicalein, confers optimal cardiac protection effects against ischemia/reperfusion injury, and this protection also involves the activation of the PTEN/AKT/NO pathway (Li et al. 2017).

## Conclusion and perspectives

**Fig. 1:**
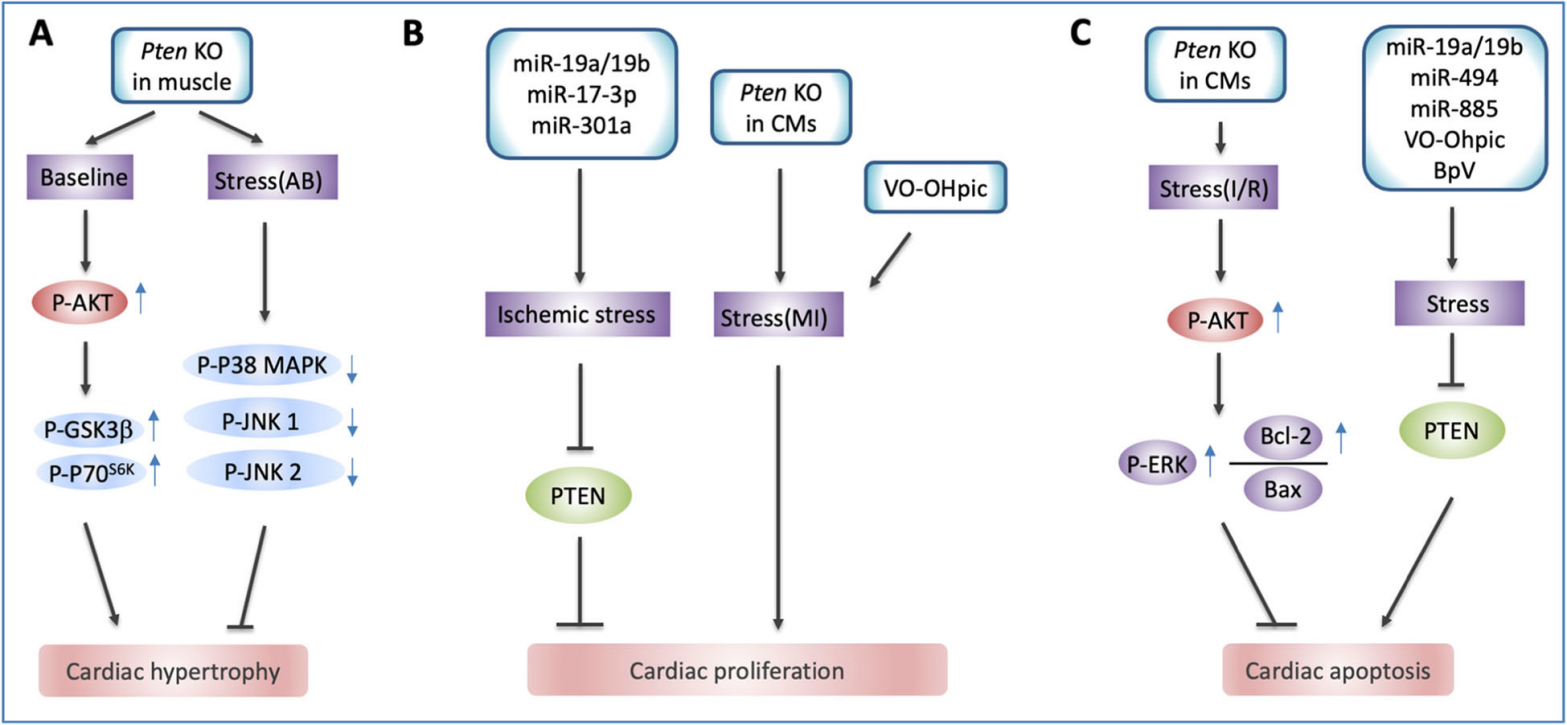
Biological processes regulated by PTEN after cardiac stress. **A**. Knockout of *Pten* with Mck-Cre induces heart hypertrophy in baseline conditions and results in reduced pathological hypertrophy in hearts subjected to aortic banding. **B**. miR-19a/19b, miR-17-3p, miR-301a, promote cardiomyocytes proliferation after ischemic stress; Cardiac specific knockout *Pten* induces cardiomyocytes proliferation after myocardial infarction; PTEN specific inhibitor VO-OHpic boosts cardiomyocytes proliferation after myocardial infarction. **C**. Cardiac inducible knockout of *Pten* in mice inhibits apoptosis signaling after ischemia/ reperfusion; miR-19a/19b, miR-494, miR-885, VO-OHpic and BpV reduces cardiomyocytes apoptosis after cardiac stress by targeting PTEN. KO, knockout; AB, aortic banding; CMs, cardiomyocytes; VO-OHpic, PTEN specific inhibitor; I/R, ischemia/reperfusion

PTEN is a tumor suppressor with highly evolutionary conservation from mouse to human. Researchers from the past two decades unveiled the critical role of PTEN-less in development, tumorigenesis, as well as in cardiac development and disease (Di Cristofano et al. 1998; Ruan et al. 2009; Stiles et al. 2004). In this review, we summarize the strategy of PTEN-less in genetic, post-transcriptional and post-translational level. Moreover, we shed light on the impact of PTEN-less in pathophysiological processes of heart in response to cardiac injury and outline the favorable role of PTEN-less for cardiac hypertrophy, regeneration, suvival and protection heart from cardiac stress (Fig. 1).

These studies highlight the notion that PTEN-less could be a potential therapeutic strategy for heart diseases, and further extend the view of cardiac regenerative medicine. Although these direct and indirect evidence indicate that PTEN-less protects heart function and enhances cardiomyocytes proliferation and regeneration after myocardial infarction injury, the underlying molecular mechanisms need to be further clearly delineated. More importantly, for ultimate clinical therapeutics, boosting cardiomyocyte proliferation and regenerating the human heart are a commendable goal, despite barely understanding of the complex process of heart regeneration for now. With development of new strategy and advanced technology, such as a high–spatiotemporal resolution examination system for genetic lineage tracing of cell proliferation (He et al. 2021), three-dimensional organoids culture skills (Li et al. 2020), single-cell analysis of cell population, combined with gene therapy and small molecule drugs, we would positively be seeing more feasible approaches explored and exploited for regenerative medicine, leading to treatment and prevention of heart disease.

## Data Availability

Not applicable.
